# Tandem oxidative amidation of benzylic alcohols by copper(II) supported on metformin-graphitic carbon nitride nanosheets as an efficient catalyst

**DOI:** 10.1038/s41598-022-07543-3

**Published:** 2022-03-10

**Authors:** Hossein Ghafuri, Mostafa Ghafori Gorab, Haniyeh Dogari

**Affiliations:** Catalysts and Organic Synthesis Research Laboratory, Department of Chemistry, Iran University of Science and Technology, 16846-13114 Tehran, Iran

**Keywords:** Catalysis, Organic chemistry

## Abstract

In this research, an efficient heterogeneous catalyst based on graphitic carbon nitride nanosheets (CN) has been reported. The CN was functionalized by 1,3-dibromopropane as a linker (CN–Pr–Br) and subsequently modified with metformin (CN–Pr–Met). Furthermore, the copper(II) was coordinated on modified CN (CN–Pr–Met–Cu(II)) and during this process, 7.94% copper(II) was loaded into the catalyst structure. The synthesized catalyst was evaluated by various techniques including fourier-transform infrared spectroscopy (FT-IR), energy dispersive X-ray spectroscopy (EDS), field emission scanning electron microscopy (FE-SEM), thermogravimetric analysis (TGA), X-ray diffraction (XRD), and inductively coupled plasma atomic emission spectroscopy (ICP-OES). CN–Pr–Met–Cu(II) was used as a catalyst in the synthesis of amides via the oxidation of benzyl alcohols. The conditions of this reaction were optimized in terms of temperature, time, amount of catalyst, type of base, oxidant, and solvent. Moreover, a variety of amides with an efficiency of 75–95% were synthesized. The reaction was carried out in the presence of benzyl alcohols, amine hydrochloride salts, tert-butyl hydroperoxide (TBHP), CaCO_3_, and CN–Pr–Met–Cu(II) at 80 °C of acetonitrile solvent. The synthesized catalyst can be easily separated from the reaction medium and reused for 7 consecutive runs without a significant reduction in reaction efficiency.

## Introduction

Carbon-based nanomaterials including carbon nanotubes, graphene, and graphitic carbon nitride (g-C_3_N_4_) have become important topics in a variety of scientific fields in recent years^1,2^. Among these materials, the g-C_3_N_4_ with features such as high chemical and thermal stability, non-toxicity, cost-effectiveness, and easy synthesis has been considered in organic synthesis and catalyst^1^, supercapacitor^3^, biosensor^4^, environment^5^, energy^6^, and medical applications^6^. The tri-s-triazine units in the structure of the g-C_3_N_4_ are one of the reasons for catalytic applications of g-C_3_N_4_^7^. In this regard, various forms of g-C_3_N_4_ including bulk^8^, nanosheets^9^, mesoporous^10^, quantum dot^11^, and nanotubes have been reported^12^. Recently, g-C_3_N_4_ in the form of nanosheets has been functionalized covalently or non-covalently by various molecules to improve its properties and applications^13^. Functionalization of g-C_3_N_4_ is usually accomplished by oxidation/carboxylation, amidation, sulfonation, phosphorylation, polymer grafting, electrostatic interaction, π–π interaction, and hydrogen bonding^13^. Also, various molecules including L-arginine^9^, ferrocene^14^, thiamine^15^, 1,4-butane sultone^16^, 5-bromovaleryl chloride^17^, 1, 3-dibromopropane have been used to functionalized g-C_3_N_4_^15^. Metformin (dimethyl biguanide) as a polydentate ligand can be also useful. Metformin is an effective drug for type 2 diabetics that lowers blood glucose levels^18^. This molecule has been used as auxiliary medicine for cancer^19^, aging^20^, and covid-19^21^. Metformin can form chelates with transition metals such as copper, nickel, palladium, and cobalt for catalytic applications^22–27^. In a study performed in 2020 by Hamed and Ali, Cu(II)–metformin was immobilized on graphene oxide as an efficient catalyst for the Beckmann rearrangement^23^. In another study performed in 2016 by Kojoori, palladium(II) was coordinated on metformin-SBA-15 and used as a catalyst in the partial hydrogenation of alkynes^26^.

Amides are an important functional group found extensively in pharmacology such as acetaminophen, cephalexin, lidocaine, diazepam, and diperodon. Also, they were used in the synthesis of polymeric materials, such as nylon, hydrogels, artificial silks, and supported catalysts. Various methods, include Staudinger reaction^28^, Beckmann rearrangement^29^, Schmidt reactions^30^, acylation of amines^31^, hydroamination of alkynes^32^, transamidation^33^, have been used to produce amides. Synthesis of amide by the mentioned methods has disadvantages such as high cost, producing a lot of wastes, low efficiency, and the use of toxic reagents. Therefore, alternative methods such as oxidative amidation of aldehydes or alcohols have been recommended^34^. Tandem oxidative amidation of benzylic alcohols was catalyzed by a series of transition metals^35–38^. Among these metals, copper(II) with low price and high biocompatibility has been considered efficient catalysts for the amidation of alcohols^39,40^. In this study, CN–Pr–Met–Cu(II) was reported as a recyclable and efficient catalyst for the one-pot conversion of benzylic alcohols to amides. The prepared catalyst contains 7.94% copper(II) and the synthesis of amides was greatly facilitated by this method. Also, this catalyst was used 7 times in the tandem oxidative amidation of benzylic alcohols without significantly reducing the efficiency of the products.

## Experimental

### Reagents and instruments

All raw materials and solvents used in this study were produced by Merck and Flucka companies and no additional purification process was performed on them. Thin Layer Chromatography (TLC) sheets containing 0.2 mm of F254 silica gel on the aluminum plates were utilized to control the reaction progress. The melting point was investigated using the Electrothermal 9100 apparatus to confirm the accuracy of products formation. FT-IR spectroscopy was used to investigate the catalyst fabrication process and confirm the formation of reaction products. This analysis was accomplished via an AVATAR device manufactured by Thermo in the range 400–4000 cm^−1^ and by KBr pellets. XRD technique was performed using PANalytical X-PERT-PRO MPD apparatus in the range of 2θ, 5° to 10° and 10° to 90° to study the crystal structures. In this analysis, Cu Kα radiation source with λ = 1.5406 Å and 2θ step size with 0.02° were utilized. FE-SEM and EDS analysis were used to study the catalyst morphology and review the catalyst components respectively, by EM8000 KYKY apparatus. TGA was done by STA504 device to verify the correctness of catalyst synthesis and its thermal stability in the temperature range of 20 °C to 1000 °C at a rate of 5 °C/min under Ar atmosphere. ICP-OES was performed using VISTA-PRO to check the recyclability and reusability of the catalyst. Preparative thin-layer chromatography (PTLC) was used to purify the reaction products, for this purpose, 20 × 20 glass plates with silica gel coating with a thickness of 0.5 mm were used. Carbon and hydrogen nuclear magnetic resonance (NMR) spectroscopy were used to evaluate the structure of some products and for this purpose VARIAN Inova 500 MHz and 125 MHz devices were used.

### Preparation of bulk g-C_3_N_4_

Bulk g-C_3_N_4_ was prepared according to an article published by Zheng et al.^41^. 10 g of melamine powder was heated in a furnace at 550 °C for 4 h under air atmosphere. Then, the obtained yellow powder was ground for further usage.

### Preparation of CN

The procedure utilized for preparation CN was introduced by Tajik et al.^9^. Initially, 2 g of as-prepared bulk g-C_3_N_4_ and 40 mL of sulfuric acid were poured into a 600 mL beaker and the mixture was stirred for 5 h at 90 °C. Then, 400 mL of ethanol was added to the stirring solution and the resulting mixture was stirred at room temperature for 2 h. After the mentioned time, the beaker was remained stationary for 48 h until the precipitate settles well. Subsequently, decantation was done and the solution was sonicated for 6 h. Finally, the precipitate was separated by centrifugation and washed with water and ethanol. The CN sheets were dried in a 60 °C oven for 24 h.

### Preparation of CN–Pr–Br

Connection of linker to CN was performed using the method reported by Rashidizadeh et al.^15^. 40 mL of dry toluene and 1 g of synthesized CN in the previous step were inserted into a 50 mL round bottom flask and placed in an ultrasonic bath for 1 h. Then, 2 mL of 1,3-dibromopropane and 1 mmol NaI were added to the mixture and refluxed for 24 h under an N_2_ atmosphere. The functionalized precipitate was separated by centrifugation and washed several times with 1 L ethanol and 0.5 L ethyl acetate to completely remove unreacted 1,3-dibromobutane from the CN surface. The final precipitate was dried overnight in an oven at 60 °C to remove residual solvent.

### Preparation of CN–Pr–Met

2.5 g of metformin hydrochloride and 3 mL of acetonitrile were poured in a round bottom flask and mixed for 30 min via a magnetic stirrer. Then 0.6 g of NaOH was added to the mixture and stirred for 1 h at room temperature. In the next step, 5 g of KI was added to the reaction mixture under extreme stirring conditions. After 30 min, 3 g of CN***–***Pr***–***Br was added to the mixture and the reaction was refluxed for 12 h. The final product was separated by centrifuge, washed with 0.5 L of methanol and 0.5 L of distilled water, and subsequently dried in an oven at 60 °C.

### Preparation of CN–Pr–Met–Cu(II)

**Figure 1:**
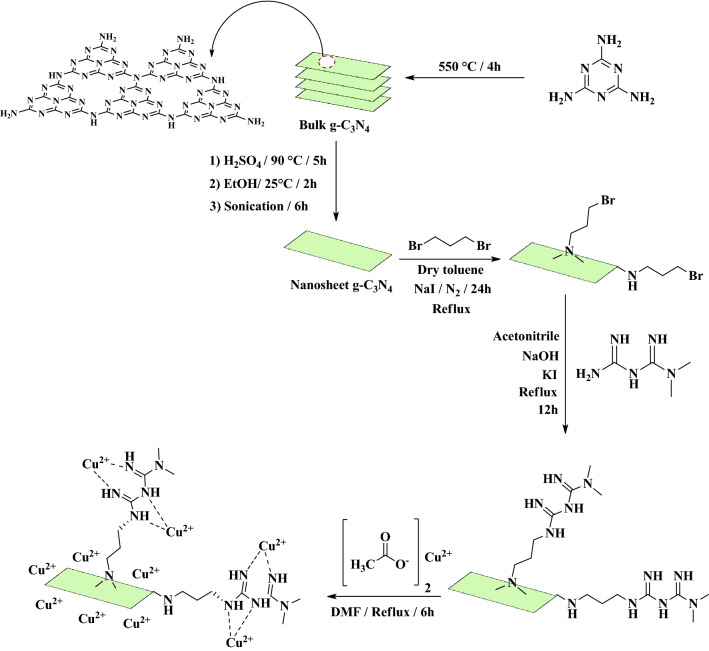
Graphical illustration of CN–Pr–Met–Cu(II) catalyst synthesis.

First, 0.5 g of CN–Pr–Met was dispersed in 40 mL of DMF. Then 0.5 g of copper(II) acetate hydrate salt was added to the solution and refluxed for 6 h. The final catalyst was collected by centrifuge and after washing with 200 mL of distilled water and 50 mL ethanol, dried in a 60 °C oven for 12 h. The catalyst synthesis procedure was shown in Fig. 1.

### General procedure for tandem oxidative amidation of benzylic alcohols via amine hydrochloride salts

1 mmol of amine hydrochloride salt, 1.5 mmol of benzyl alcohol derivatives, 20 mg of catalyst, 3 mL of acetonitrile, 1.1 equivalents of calcium carbonate, and 4 equivalents of TBHP are mixed. The reaction mixture was stirred under atmospheric nitrogen for 3 h at 80 °C and the reaction progress was controlled by TLC. After this time, the catalyst was collected by using filter paper, and on the other hand, the filtrate was extracted with water and dichloromethane. The amide precipitates in dichloromethane were formed by using the anti-solvent crystallization method. Finally, the precipitate was purified using PTLC.

## Result and discussion

In this research, a novel heterogeneous catalyst was synthesized via functionalization of CN for the tandem oxidative amidation of benzylic alcohols. Figure 1 was shown the preparation of CN–Pr–Met–Cu(II). Initially, bulk g-C_3_N_4_ was converted to a CN by chemical exfoliation procedure. In the next step, during a nucleophilic reaction, 1,3-dibromopropane as a linker was attached to the CN. Subsequently, metformin was connected to the linker by a covalent bond, and copper(II) was coordinated to metformin and CN. The as-prepared catalyst was used for the amide synthesis reaction by oxidation of benzylic alcohols in the presence of amine hydrochloride salts. The reaction was investigated in the presence of benzylic alcohol derivatives with electron donor and withdrawing groups as well as different types of amine salts. Furthermore, various analyzes include FT-IR, EDS, XRD, TGA, FE-SEM, and ICP-OES were used to confirm the structure of the catalyst.

### Catalyst characterizations

#### FT-IR analysis

**Figure 2:**
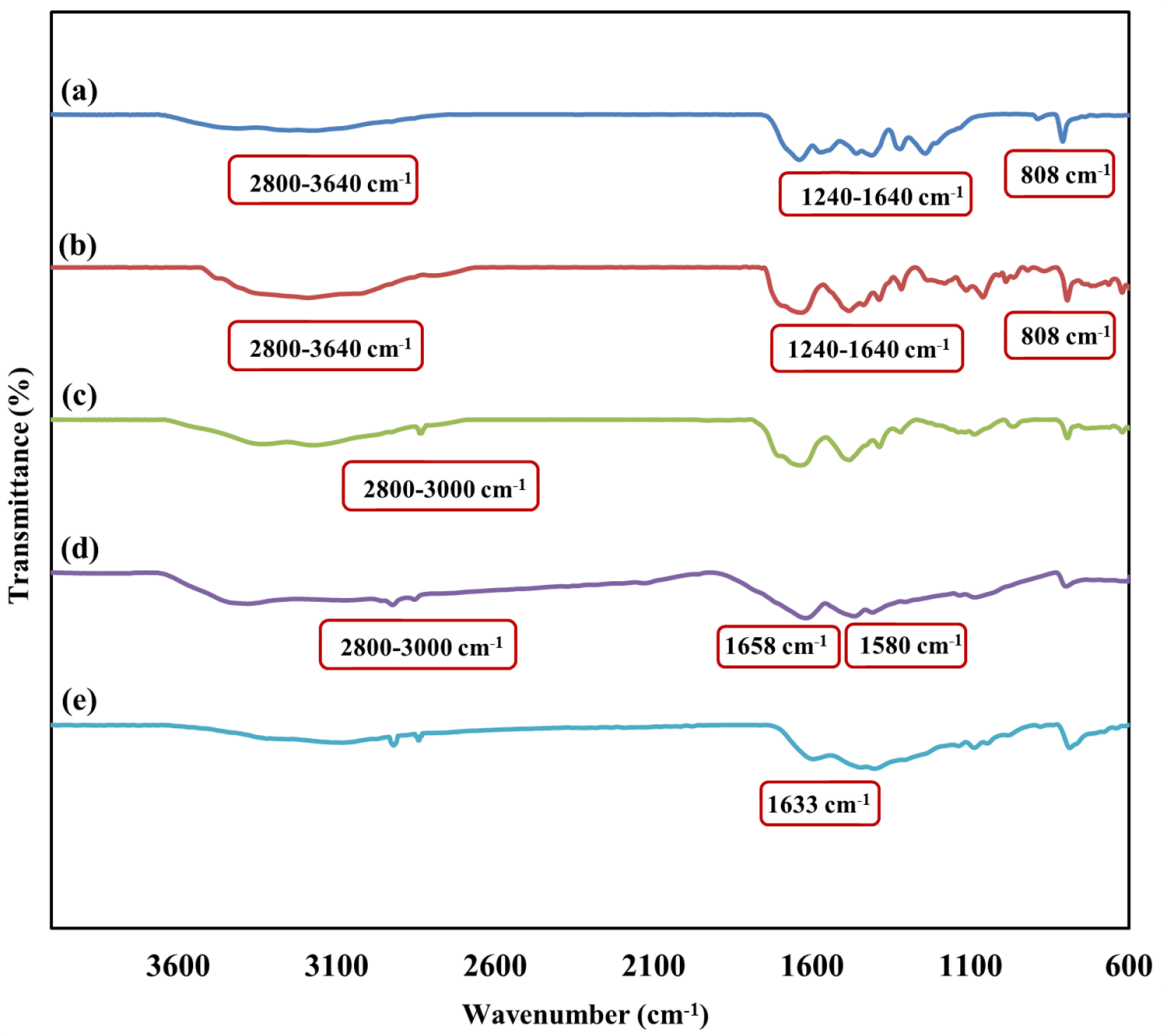
FT-IR spectrum of (a) bulk g-C_3_N_4_, (b) CN, (c) CN–Pr–Br, (d) CN–Pr–Met and (e) CN–Pr–Met–Cu(II).

FT-IR spectrum of bulk g-C_3_N_4_ was shown in Fig. 2a. a broad peak was observed in the range of 2800 cm^−1^ to 3640 cm^−1^, which corresponds to the N–H groups of the molecule, including the –NH_2_ and =NH groups^42^. Strong peaks in the region 1240 cm^−1^ to 1640 cm^−1^ were identified due to the stretching vibrations of heterocyclic C–N and C=N respectively, and the peak observed in 808 cm^−1^ was related to triazine units^42^. Figure 2b was described the CN. Approximately all peaks in the FT-IR spectrum of bulk g-C_3_N_4_ are also present in the CN, but the slight difference in their intensity is due to the protonation of bulk g-C_3_N_4_ by H_2_SO_4_^43,44^. In Fig. 2c, alongside the absorbance bands of the g-C_3_N_4_, the peak appears in the range of 2800 cm^−1^ to 3000 cm^−1^ was associated with the stretching vibration of the aliphatic C–H in the linker^9^. Figure 2d illustrates the functionalization of CN–Pr–Br by metformin. The peak in 2940 cm^−1^ was demonstrated the stretching vibration of the C–H in the CH_3_ group of the metformin^23^. Moreover, the peak that observes approximately in 1658 cm^−1^ and 1580 cm^−1^ may be related to the stretching vibration of the C=NH and N–H respectively^45^. According to Fig. 2e which was shown the CN–Pr–Met–Cu(II), the C=NH peak of the metformin shifted to the lower frequencies of the spectrum and proves the coordination of copper(II) to metformin.

#### EDS analysis

**Figure 3:**
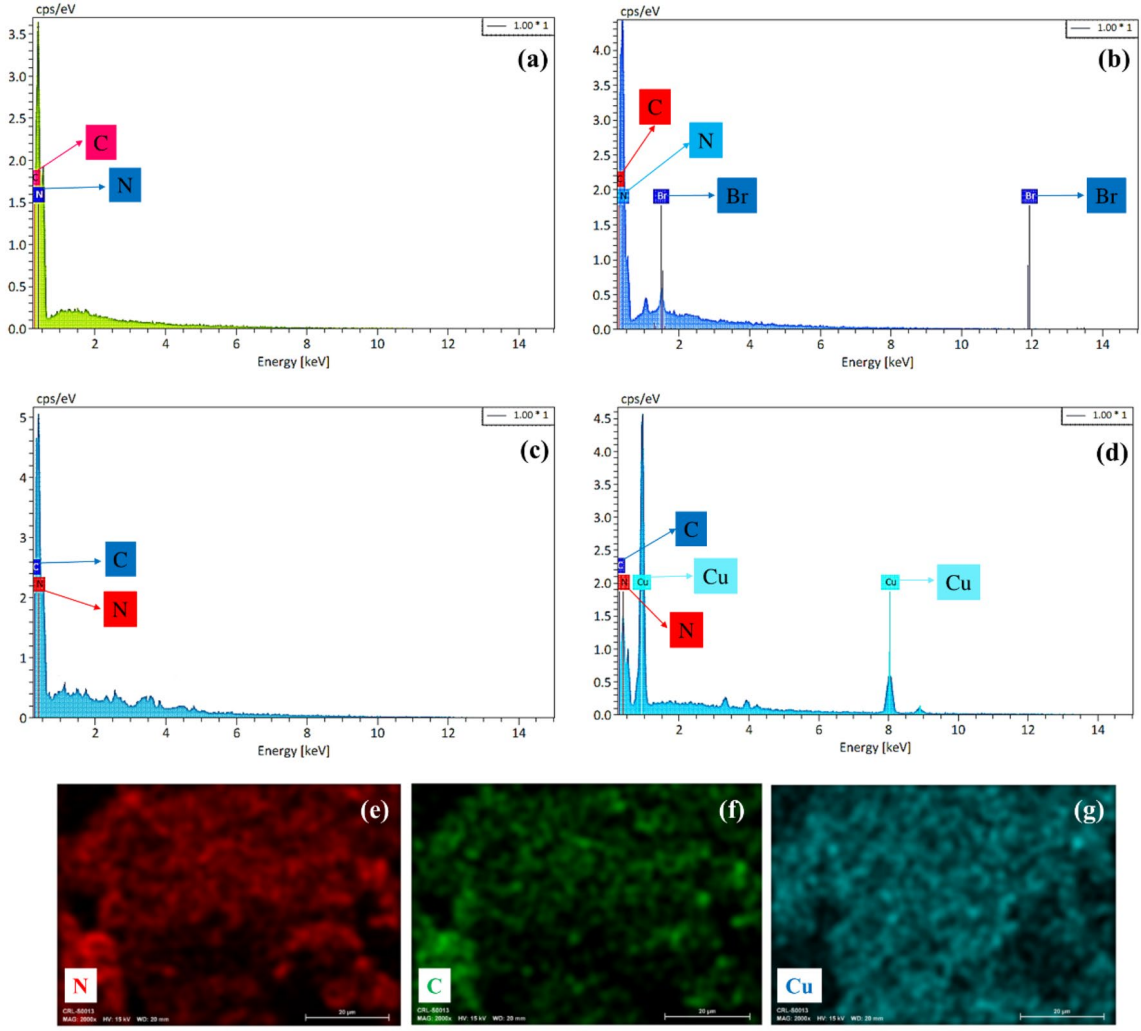
EDS spectrum of (**a**) CN, (**b**) CN–Pr–Br, (**c**) CN–Pr–Met, (**d**) CN–Pr–Met–Cu(II) and (**e–g**) elemental mapping of the CN–Pr–Met–Cu(II).

EDS analysis from different stages of catalyst synthesis was shown in Fig. 3. Based on Fig. 3a,b, the presence of carbon and nitrogen in the structure of the CN and its functionalization by 1,3-dibromopropane via the presence of bromine was confirmed. Figure 3c has shown the EDS analysis of CN functionalized by metformin. Due to the similar elements in the structure of metformin and CN, carbon and nitrogen are visible in the EDS spectrum, and the remarkable point is the removal of bromine, which confirms the binding of metformin to the CN–Pr. In Fig. 3d, the presence of C, N and Cu elements was confirmed in the CN-Pr–Met–Cu(II). In addition, EDS mapping of CN–Pr–Met–Cu(II) demonstrated excellent dispersion of C, N, and Cu elements in the structure (Fig. 3e–g).

#### FE-SEM imaging

**Figure 4:**
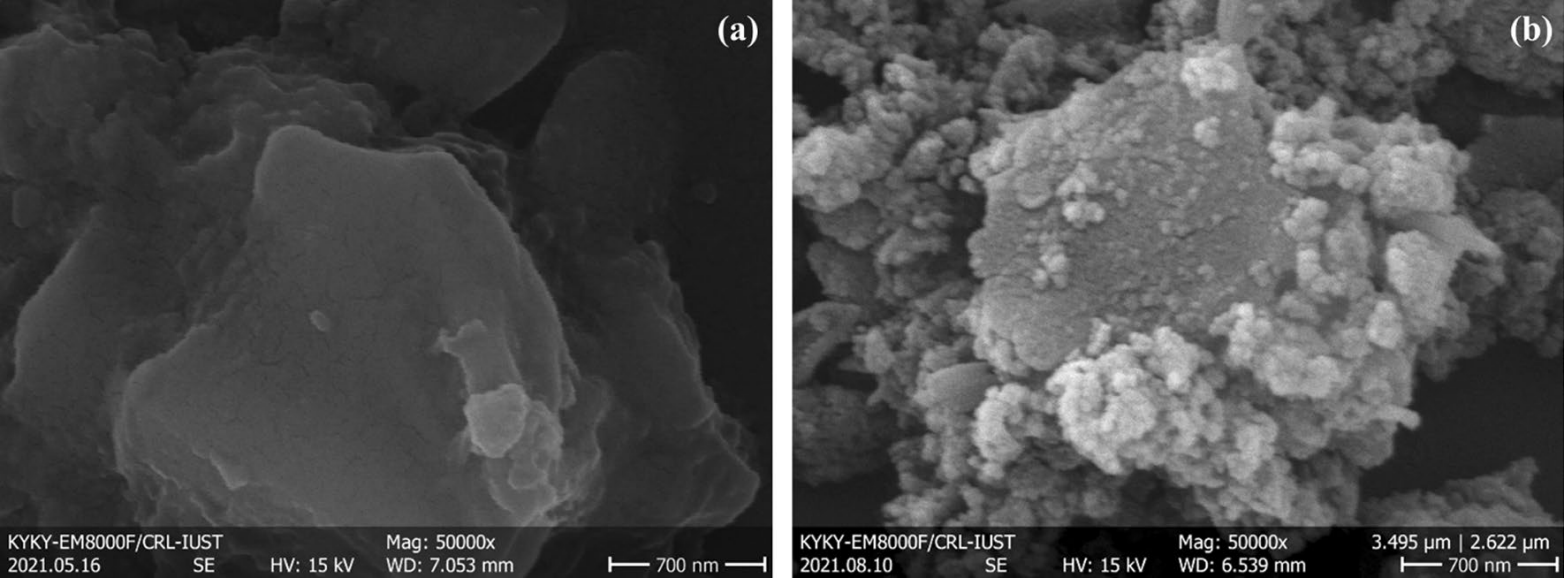
FE-SEM image of (**a**) CN and (**b**) CN–Pr–Met–Cu(II).

FE-SEM imaging was evaluated in Fig. 4 to study the morphology of synthesized CN and CN–Pr–Met–Cu(II). According to Fig. 4a,b, the sheets in the structure of CN and change during the functionalization process, suggesting that surface of the CN sheets were covered by synthesized ligand.

#### XRD analysis

**Figure 5:**
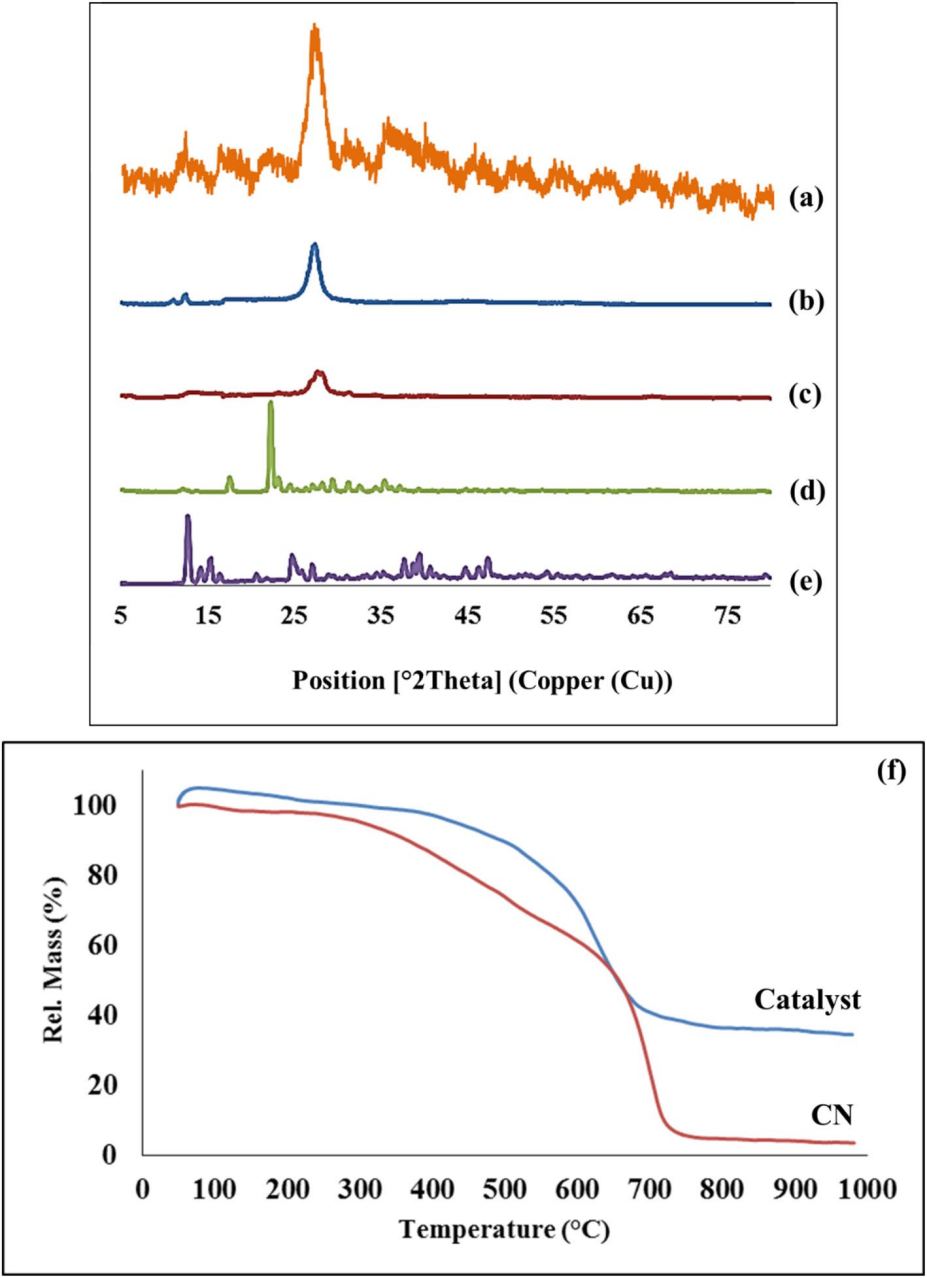
XRD pattern of (a) prepared CN–Pr–Met–Cu(II), (b) bulk g-C_3_N_4_, (c) CN, (d) Metformin, and (e) Cu(II) acetate hydrate. (f) TG analysis of the CN–Pr–Met–Cu(II) and CN.

The XRD pattern of the synthesized CN–Pr–Met–Cu(II) was shown in Fig. 5a. Peaks related to CN, metformin, and Cu(II) acetate hydrate were observed in the XRD pattern of the prepared catalyst. XRD pattern of bulk g-C_3_N_4_ was shown in Fig. 5b. The peak observed in 2θ about 26.8° is related to the interplanar stacking of the conjugated aromatic systems for (002) reflection. Also, the observed peak in 2θ about 13.1° corresponds to the in-plane structural packing motif of repeated tri-s-triazine units for (100) reflection^15,43^. Comparing the XRD pattern in Fig. 5b,c shows that bulk g-C_3_N_4_ has been converted to CN. After exfoliation, the (002) peak intensity was decreased, and on the other hand, this peak appeared at a slightly higher diffraction angle^15^. Moreover, the peak at 2θ about 13.1° has almost disappeared. These observations are due to the shortening of the interplanar stacking space in CN^15^. Observation of all metformin peaks at 2θ around 12.2°, 17.7°, 22.4°, 24.5°, 31.1°, and 39.4° can confirm the maintenance of the crystalline structure of the metformin in the final catalyst (Fig. 5d)^46^. Cu(II) acetate hydrate peaks are also visible in the final CN–Pr–Met–Cu(II) pattern in 2θ around 12.75°, 14.2°, 15.37°, 15.53°, 16.37°, 25.9, 37.81°, 38.82°, 39.4°, 40.79°,46.35°, 47.37°, and 48.16° (Fig. 5e).

#### TG analysis

The thermal stability of the catalyst and CN were investigated by TG analysis in Fig. 5f. The synthesized catalyst was stable up to 400 °C, although its partial mass loss in the range of 50 °C to 100 °C was due to the evaporation of adsorbed water or air contaminants collected by the solids. The observed mass reduction in the range of 100 °C to about 500 °C can be related to the destruction of organic parts and thermal deformation/collapse of the CN^23,47^. The CN–Pr–Met–Cu(II) main mass loss occurred at 500 °C to 700 °C, which was related to the thermal decomposition or deformation of heptazine units in the structure of the CN^47^. The CN–Pr–Met–Cu(II) has higher thermal stability than CN, and this may be due to the synergistic effect and supramolecular interactions between carbon nitride and other components of the catalyst^48^.

#### Catalytic properties

**Figure 6:**
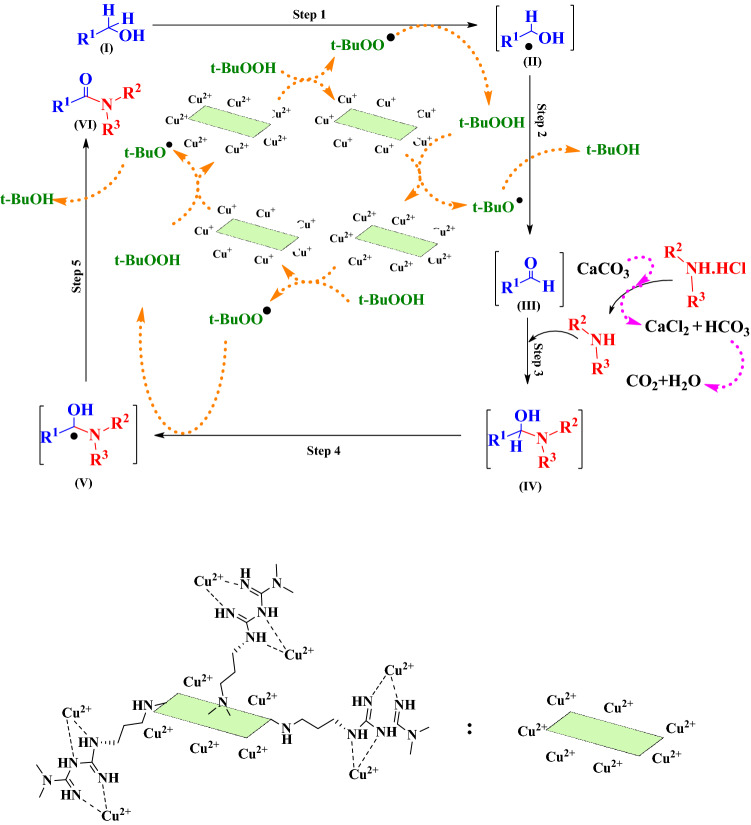
Proposed mechanism for tandem oxidative amidation reaction performed using the novel CN–Pr–Met–Cu(II) catalyst.

The catalytic performance of CN–Pr–Met–Cu(II) was explored for the tandem oxidative amidation of benzylic alcohols. In this regard, optimization was performed to evaluate the best conditions for the reaction (Table 1). Initially, the reaction was performed in the presence of 20 mg of CN–Pr–Met–Cu(II) catalyst, with 1.1 equivalent CaCO_3_ as a base and 4 equivalent TBHP as oxidant at ambient temperature under inert N_2_ atmosphere. In this case, 35% yield of the product was observed (Table 1, entry 1). Then, the effect of temperature, solvent, oxidant, base, the amount of catalyst, and time were examined. Temperatures from 25 to 120 °C were tested (Table 1, entries 1–6). With the increasing temperature up to 80 °C, the reaction efficiency increases, but in the next step with increasing temperature up to 120 °C, a decrease in reaction efficiency was observed. This observation is due to the oxidation of benzyl alcohols to benzoic acid^49^. In addition, the reaction was checked in different amounts of CN–Pr–Met–Cu(II) and it was observed that the optimized amount of catalyst was 20 mg (Table 1, entries 4, 8–9). Subsequently, the reaction was checked in the presence of CaCO_3_, K_2_CO_3_, and Na_2_CO_3_ as a base and the CaCO_3_ has the best performance (Table 1, entries 4, 10–11). Low solubility and weak basicity of CaCO_3_ caused slow deprotonation of the amine hydrochloride salt. Bases such as K_2_CO_3_ and Na_2_CO_3_, by rapid deprotonation of the amine hydrochloride salt and the immediate production of free amines, cause the formation of undesirable intermediates (imine) instead of the intermediate (IV) (Fig. 6). Therefore, the use of K_2_CO_3_ and Na_2_CO_3_ reduced the reaction yield^50^. The effect of solvents (Table 1, entries 4, 12–13) and oxidants (Table 1, entries 4, 14–15) was evaluated and the best result was provided in acetonitrile solvent by TBHP. Then for optimization reaction time, the reaction was carried out in 2 to 6 h, and the best efficiency was provided in about 3 h (Table 1, entries 4, 16–18). The reaction did not carry out by removing each of the catalyst, oxidant, and base (Table 1, entries 19–21). In addition in Table 2, to confirm the performance synthesized catalyst and the effect of its components in the reaction, CN (Table 2, entry 1), copper(II) acetate hydrate (Table 2, entry 2), metformin (Table 2, entry 3), a mixture of metformin and copper(II) acetate hydrate (1:1) (Table 2, entry 4), a mixture of CN and copper(II) acetate hydrate (1:1) (Table 2, entry 5), CN–Pr–Br (Table 2, entry 6), CN–Pr–Met (Table 2, entry 7), and CN–Pr–Met–Cu(II) (Table 2, entry 8) were added separately to the reaction as a catalyst and the efficiency of the obtained product was evaluated. As predicted, CN–Pr–Met–Cu(II) catalyst had a higher ability to perform the reaction. Based on the results, it was determined that the main component to catalyze the reaction is copper ions. Table 3 summarizes the products synthesized by CN–Pr–Met–Cu(II) catalyst. The formation of products was confirmed by melting point. Moreover, FT-IR, ^1^H-NMR and ^13^C-NMR of selected amides are shown in the supplementary information file (Figs. S1–S6).

**Table 1:** Optimization of the reaction condition for the one-pot direct oxidative amidation.


Entry	Solvent	Oxidant	Base	Amount of catalyst (mg)	Time (h)	Temperature (°C)	Yield (%)^a^
1	CH_3_CN	TBHP	CaCO_3_	20	3	25	35
2	CH_3_CN	TBHP	CaCO_3_	20	3	40	43
3	CH_3_CN	TBHP	CaCO_3_	20	3	60	65
**4**	**CH**_**3**_**CN**	**TBHP**	**CaCO**_**3**_	**20**	**3**	**80**	**95**
5	CH_3_CN	TBHP	CaCO_3_	20	3	100	78
6	CH_3_CN	TBHP	CaCO_3_	20	3	120	70
8	CH_3_CN	TBHP	CaCO_3_	10	3	80	65
9	CH_3_CN	TBHP	CaCO_3_	30	3	80	95
10	CH_3_CN	TBHP	K_2_CO_3_	20	3	80	Trace
11	CH_3_CN	TBHP	Na_2_CO_3_	20	3	80	Trace
12	CH_3_CN	H_2_O_2_	CaCO_3_	20	3	80	30
13	CH_3_CN	O_2_	CaCO_3_	20	3	80	15
14	DMF	TBHP	CaCO_3_	20	3	80	50
15	DMSO	TBHP	CaCO_3_	20	3	80	65
16	CH_3_CN	TBHP	CaCO_3_	20	5	80	95
17	CH_3_CN	TBHP	CaCO_3_	20	6	80	95
18	CH_3_CN	TBHP	CaCO_3_	20	2	80	85
19	CH_3_CN	TBHP	CaCO_3_	–	3	80	–
20	CH_3_CN	–	CaCO_3_	20	3	80	–
21	CH_3_CN	TBHP	–	20	3	80	–

Reaction condition: benzylamine hydrochloride (1.0 mmol), benzyl alcohol (1.5 mmol), catalyst (CN–Pr–Met–Cu(II)), solvent (3.0 mL), base (1.1 Equiv), oxidant (70 wt % in H_2_O, 4 Equiv), under N_2_ atmosphere.

^a^Isolated yield.

**Table 2:** A comparison of the catalytic efficiency of CN–Pr–Met–Cu(II) and its components in tandem oxidative amidation of benzylic alcohols.

**Entry**	Catalyst	Yield (%)^a^
1	CN	–
2	Copper (II) acetate hydrate	10
3	Metformin	–
4	Copper (II) acetate hydrate + Metformin (1:1)	35
5	CN + Copper (II) acetate hydrate (1:1)	60
6	CN–Pr–Br	–
7	CN–Pr–Met	–
8	CN–Pr–Met–Cu(II)	95

Reaction condition: benzylamine hydrochloride (1.0 mmol), benzyl alcohol (1.5 mmol), catalyst (20 mg), CH_3_CN (3 mL), CaCO_3_ (1.1 Equiv), TBHP (70 wt % in H_2_O, 4 Equiv).

^b^Isolated yield.

**Table 3:** Direct oxidative amidation of benzyl alcohols with amine hydrochloride salts.

Entry	Alcohol	Amine salt	Product	Yield (%)^a^	Mp. (°C)	
					Found	Reported
1				90	105	104–105^52^
2				95	111	110–111^53^
3				95	120	120–122^54^
4				80	163	162–164^55^
5				80	140	140–142^56^
6				80	97	95–97^49^
7				90	127	125–127^57^
8				95	116	115–116^58^
9				95	126	126–128^59^
10				75	192	192–194^60^
11				80	168	167–170^61^
12				75	73	73–75^62^
13				80	132	132–134^63^
14				75	101	100–102^64^

Reaction condition: amine hydrochloride (1.0 mmol), benzyl alcohols (1.5 mmol), catalyst (CN–Pr–Met–Cu(II)) (20 mg), CH_3_CN (3 mL), CaCO_3_ (1.1 Equiv), TBHP (70 wt % in H_2_O, 4 Equiv).

^a^Isolated yield.

**Figure 7:**
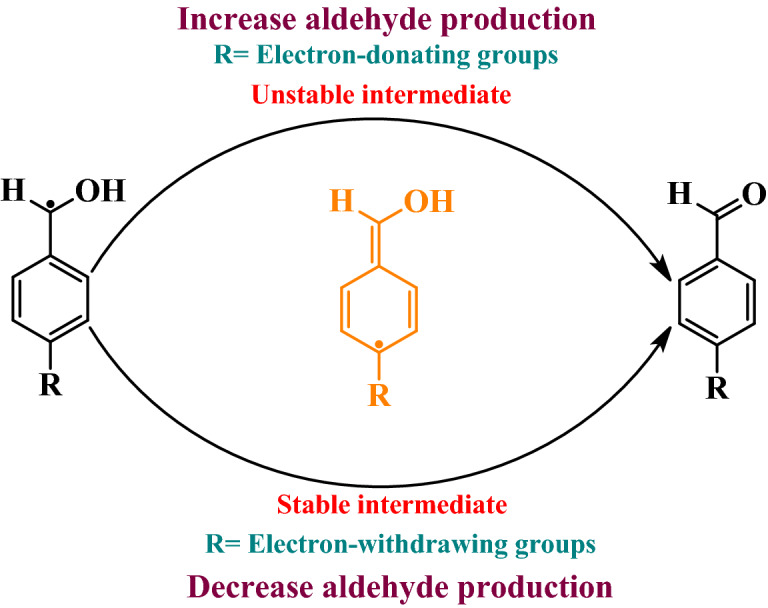
The effect of electron-withdrawing and electron-donating groups on aldehyde formation.

Various benzylic alcohols and amines were used to synthesize amides via a tandem oxidative amidation reaction. In this regard, benzyl alcohols with donor groups (CH_3_, and OCH_3_) and withdrawing groups (Cl, Br, and NO_2_), respectively increase and decrease the product’s efficiency. Benzylic alcohols with electron-withdrawing groups; caused the stability of the radical intermediate during the conversion of alcohol to aldehydes and reduce aldehyde production. On the other hand, electron donor groups on the benzylic alcohols made the intermediate unstable and increases its conversion to aldehydes (Fig. 7)^51^. In addition, the efficiency was not significantly different in the presence of aromatic and aliphatic amines.

According to the previous studies and observed results, the radical mechanism has been proposed for this reaction (Fig. 6)^65^. In step 1, intermediate (II) was formed during the separation of a hydrogen from benzyl alcohol (I). In step 2, intermediate (II) was converted to an aldehyde (III) by losing hydrogen and aldehyde formation was confirmed using TLC^66^. In step 3, the aldehyde reacts with the amine in the presence of CaCO_3_ and forms the hemiaminal intermediate (IV)^67^. Subsequently in step 4, tert-butylperoxyl radical was produced by the reaction of the catalyst with TBHP oxidant. The active tert-butylperoxyl radical separates the hydrogen from the hemiaminal (IV) and formed the intermediate (V). Finally, in step 5, the desired amide (VI) was formed via oxidation of intermediate (V). A radical scavenger was used to confirm the radical mechanism. In this regard, 1 equivalent of 2,6-di-t-butyl-4-methylphenol (BHT) was added to the model reaction and no product was formed^49^.

**Figure 8:**
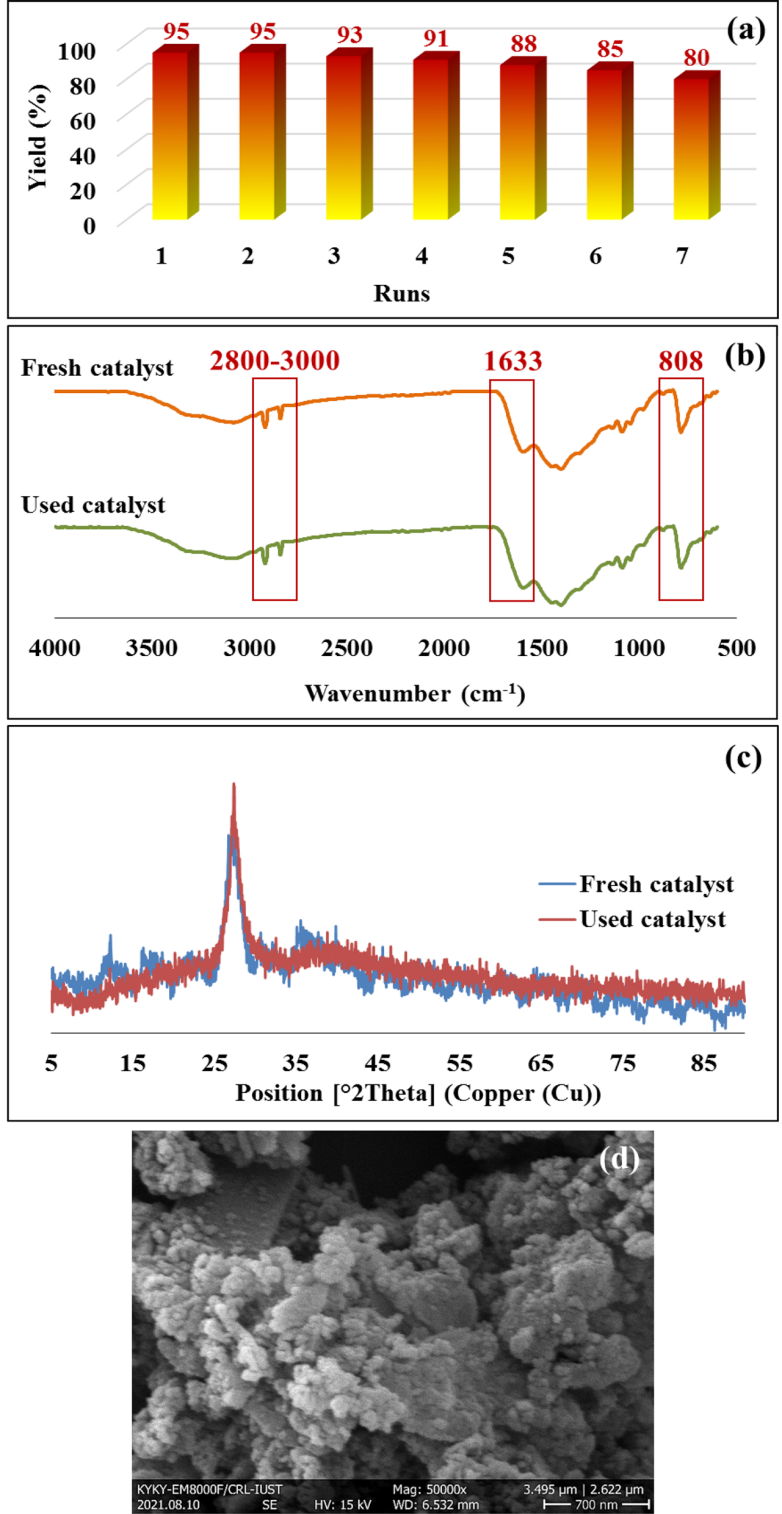
(**a**) Recyclability of the CN–Pr–Met–Cu(II) in tandem oxidative amidation of benzyl alcohol with benzylamine hydrochloride salt. (**b**) FT-IR, (**c**) XRD analysis of fresh and used CN–Pr–Met–Cu(II). (**d**) FE-SEM image of used CN–Pr–Met–Cu(II).

In addition, the reusability of the CN–Pr–Met–Cu(II) was evaluated under the optimized reaction. The efficiency of the catalytic reaction did not decrease significantly after seven reaction cycles (Fig. 8a). Also, FT-IR, FE-SEM, and XRD results indicated that the structure of the catalyst has not changed (Fig. 8b–d). Catalyst leaching was also evaluated and according to the obtained results from ICP-OES analysis, Cu% decreased from 7.94 to 7.88% after seven cycles.

Following the investigation of the catalyst properties from different points of view, a comparison was made between the performance of the synthesized catalyst and the catalysts reported in the literature. According to Table 4, CN–Pr–Met–Cu(II) was acceptable in terms of efficiency, recyclability, and reaction time.

**Table 4:** Comparison of the catalytic ability of CN–Pr–Met–Cu(II) with previous reports for amides synthesis via the oxidative amidation of benzylic alcohols.

Entry	Catalyst	Oxidizing agent	Base	Reaction time (h)	Yield (%)	References
1	KI-TBHP	–	–	15	77	^68^
2	Au/DNA	O_2_	LiOH·2H_2_O	12	80	^69^
3	Fe_3_O_4_@Fe(OH)_3_	TBHP	CaCO_3_	6	89	^65^
4	CuO	TBHP	CaCO_3_	4	70	^40^
5	Au6Pd/resin	O_2_	NaOH	12	83	^70^
6	Diacetoxyiodobenzene	TBHP	–	10	69	^71^
7	Fe(NO_3_)_3_	O_2_/TBHP	CaCO_3_	16	78	^72^
8	(PyPS)_3_PW_12_O_40_	TBHP	–	12	80	^73^
9	Ompg-C_3_N_4_/Cu	TBHP	CaCO_3_	4	93	^49^
10	CN–Pr–Met-Cu(II)	TBHP	CaCO_3_	3	95	This work

## Conclusion

In this study, we synthesized a promising heterogeneous catalyst made of CN, metformin, 1,3-dibromopropane, and copper(II) acetate hydrate. The CN–Pr–Met–Cu(II) catalyst exhibited distinguished catalytic activity for tandem oxidative amidation of benzylic alcohols in the presence of amine hydrochloride salt. Amides with good to excellent yields were synthesized in 3 h with 0.02 g of CN–Pr–Met–Cu(II) catalyst. Moreover, the fabricated catalyst showed high thermal resistance, and about 40% of the catalyst weight was maintained up to 1000 °C. The recyclability of CN–Pr–Met–Cu(II) catalyst is a remarkable feature that could be reused 7 times without a significant reduction in its efficiency.

## Supplementary Information

Supplementary Figures.
