# Supplementary material for: Tandem oxidative amidation of benzylic alcohols by copper(II) supported on metformin-graphitic carbon nitride nanosheets as an efficient catalyst
Source: Sci Rep. 2022 Mar 10;12:4221. doi: 10.1038/s41598-022-07543-3 (PMC8908756; doi:10.1038/s41598-022-07543-3)
Supplement: Supplementary file 1 — Supplementary Figures. [file 41598_2022_7543_MOESM1_ESM.docx]

**Supplementary information**

**Tandem oxidative amidation of benzylic alcohols by copper (II) supported on metformin-graphitic carbon nitride nanosheets as an efficient catalyst**

Hossein Ghafuri*, Mostafa Ghafori Gorab, Haniyeh Dogari

*Catalysts and Organic Synthesis Research Laboratory, Department of Chemistry, Iran University of Science and Technology, Tehran 16846-13114, Iran*

**Corresponding author. E-mail: ghafuri@iust.ac.ir*

| Table of contents | | |
| --- | --- | --- |
| Entry | **Subject** | **Page** |
| 1 | Fig. S1. FT-IR spectrums of preparated *N*-benzylbenzamide. | S3 |
| 2 | Fig. S2. H-NMR spectrums of preparated *N*-benzylbenzamide. | S4 |
| 3 | Fig. S3. C-NMR spectrums of preparated *N*-benzylbenzamide. | S5 |
| 4 | Fig. S4. FT-IR spectrums of preparated benzamide. | S6 |
| 5 | Fig. S5. H-NMR spectrums of preparated benzamide. | S7 |
| 6 | Fig. S6. C-NMR spectrums of preparated benzamide. | S8 |


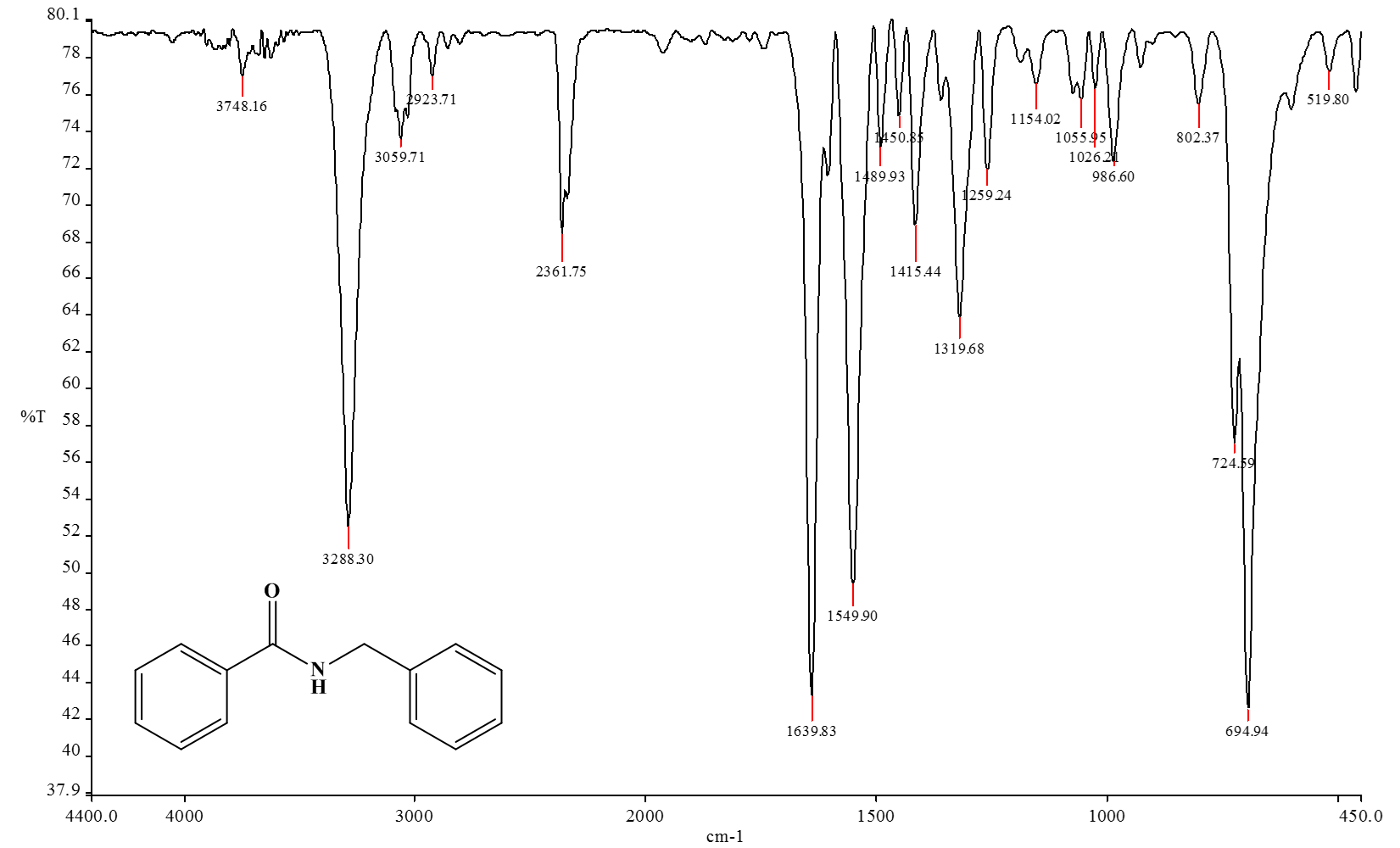


**Fig. S1.** FT-IR spectrums of preparated *N*-benzylbenzamide.


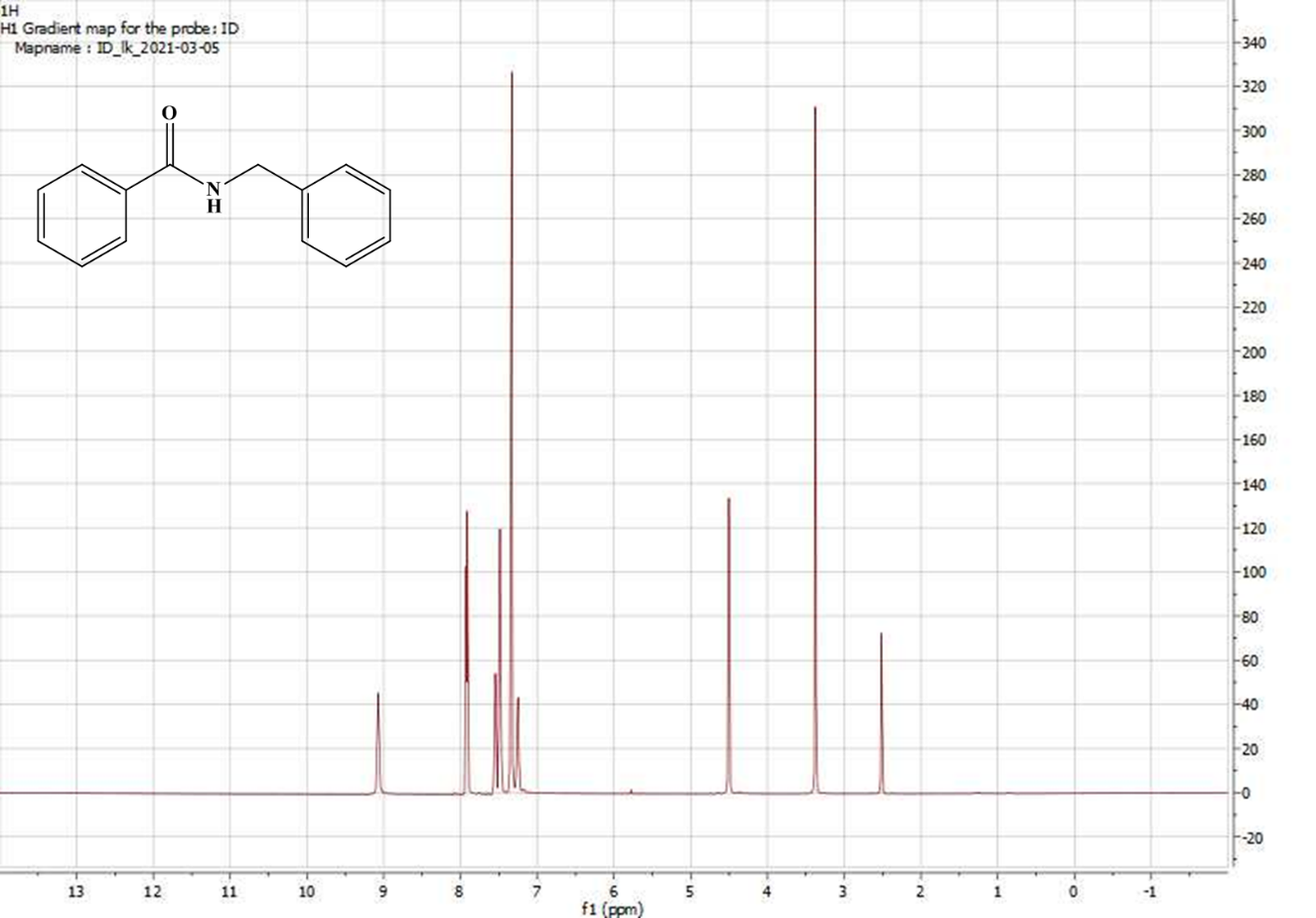


**Fig. S2.** H-NMR spectrums of preparated *N*-benzylbenzamide.


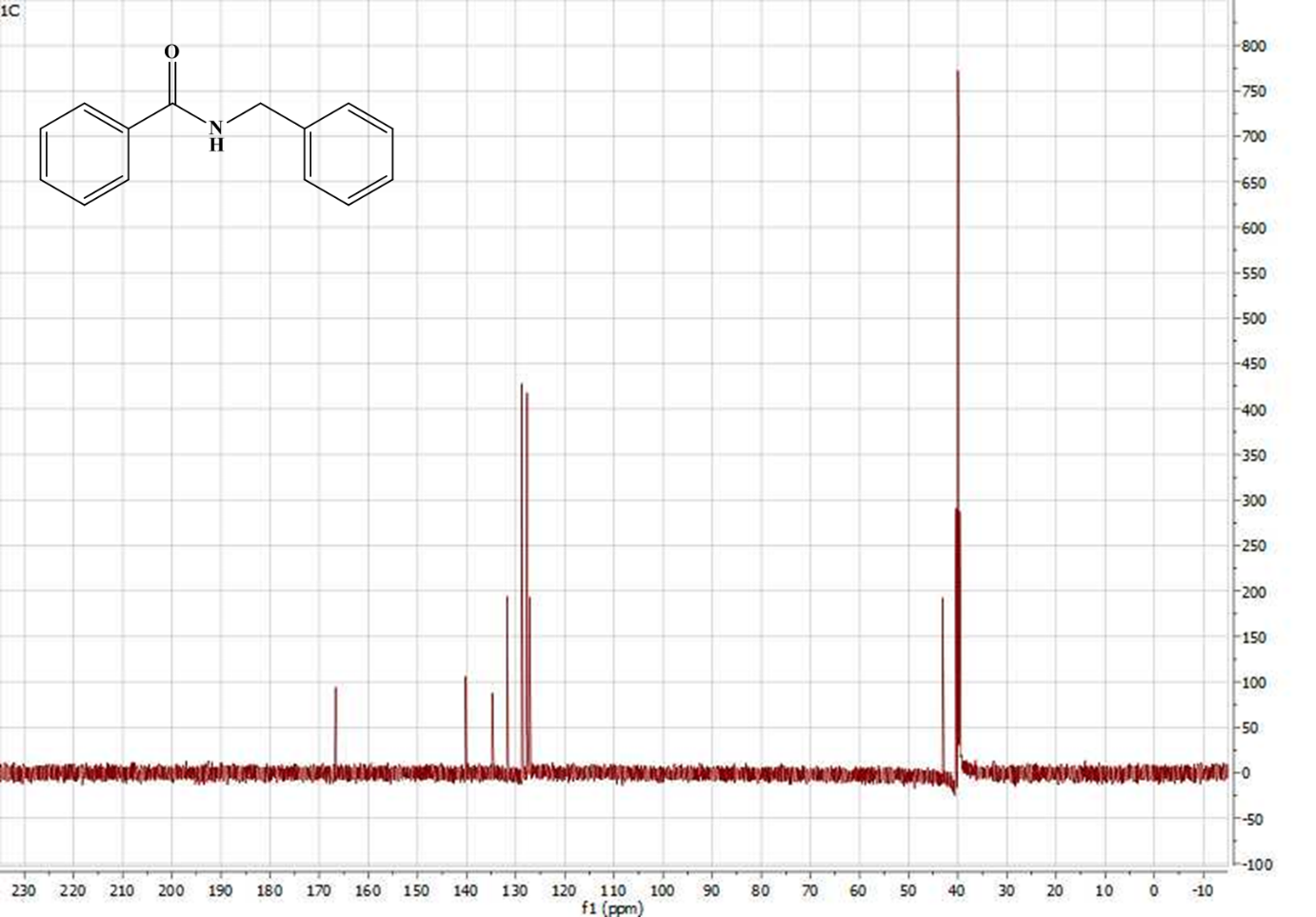


**Fig. S3.** C-NMR spectrums of preparated *N*-benzylbenzamide.


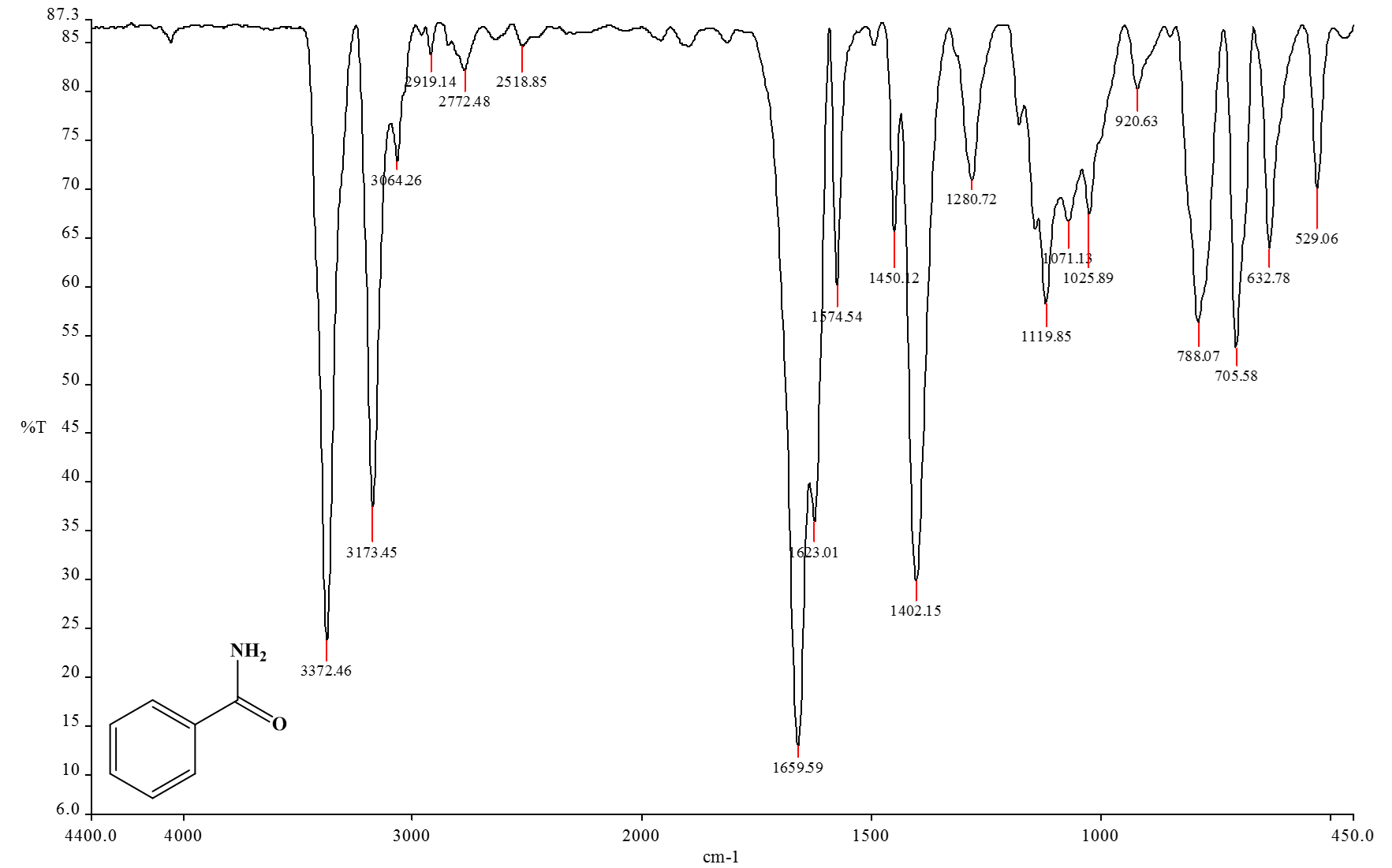


**Fig. S4.** FT-IR spectrums of preparated benzamide.


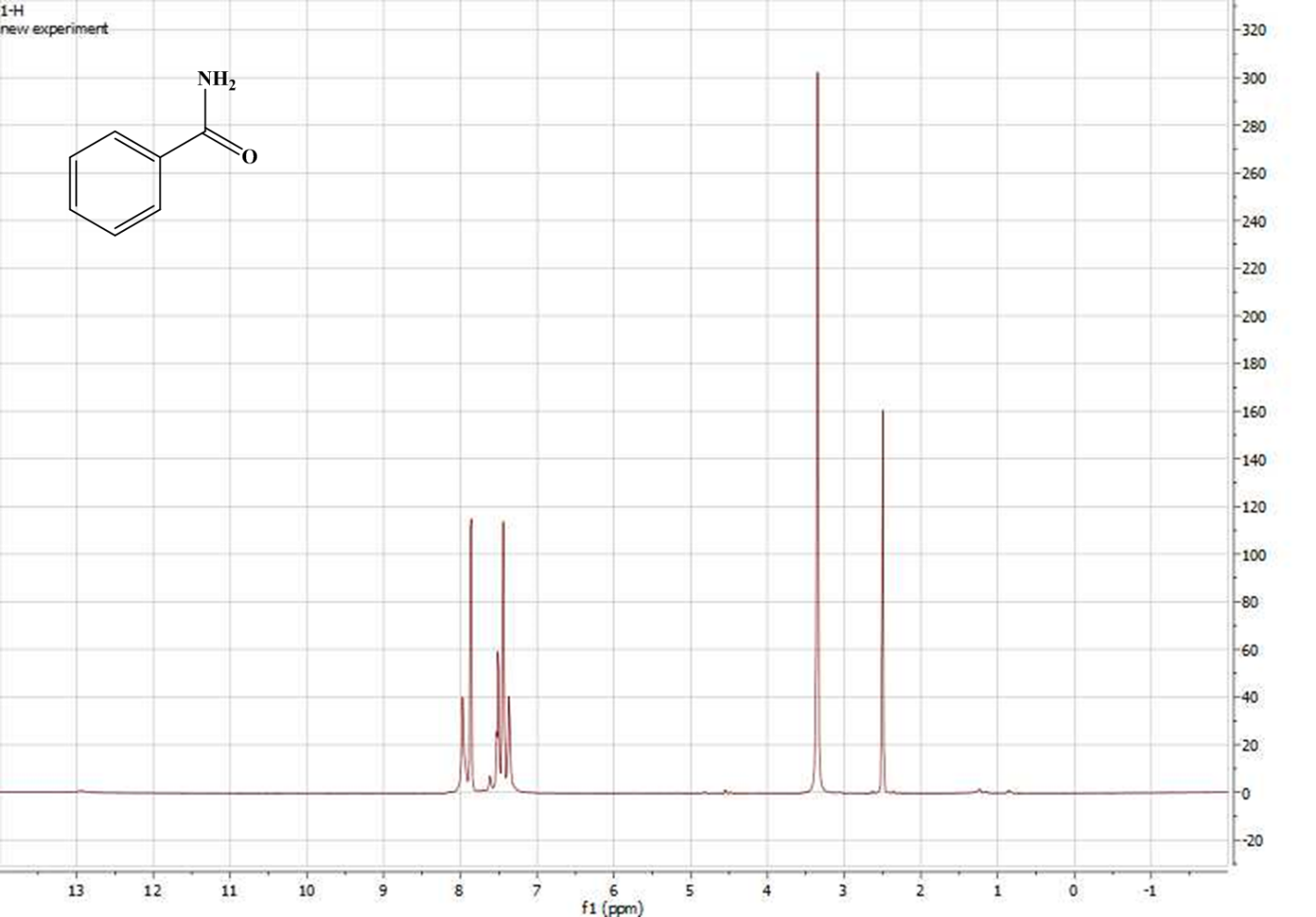


**Fig. S5.** H-NMR spectrums of preparated benzamide.


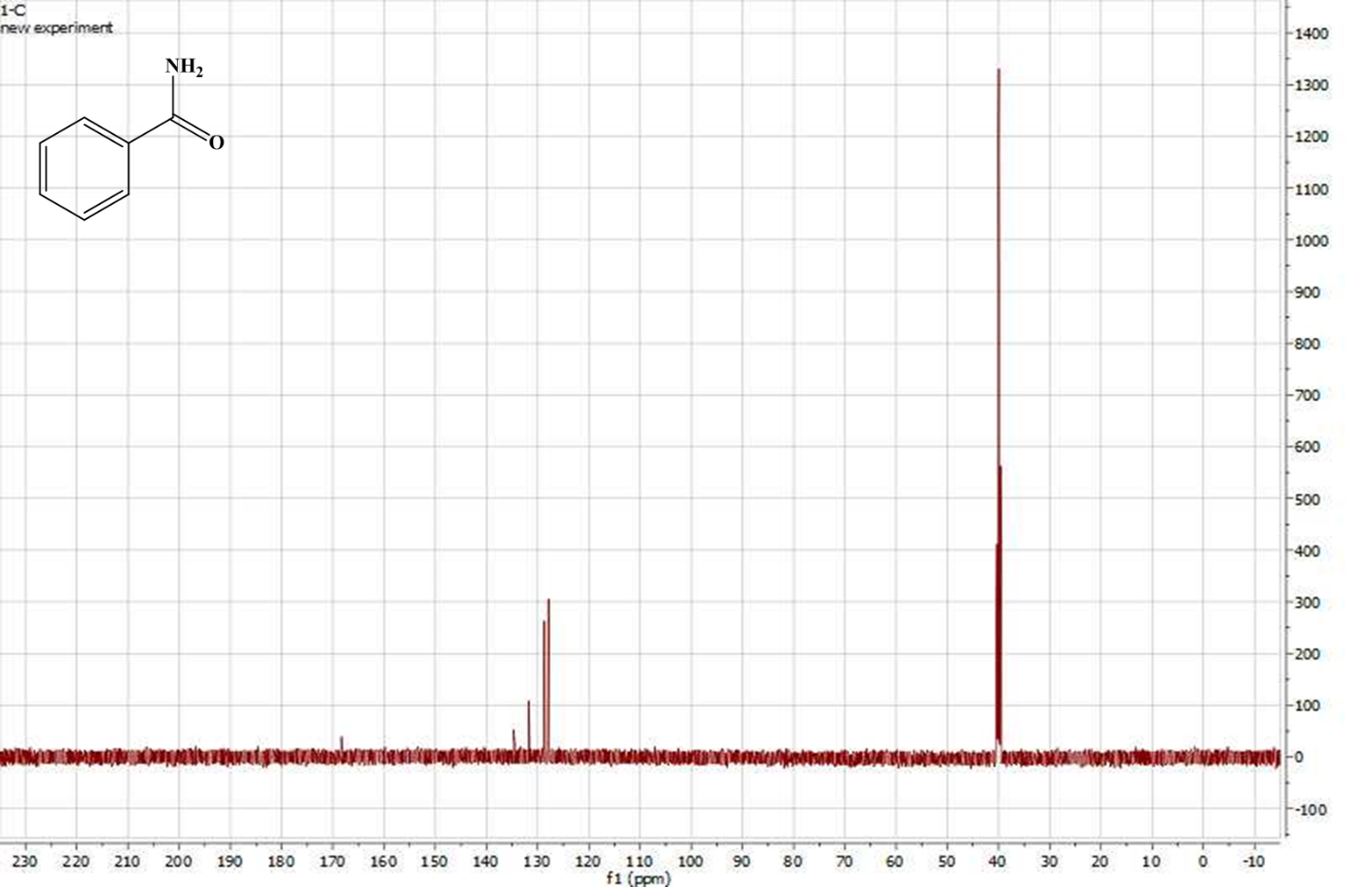


**Fig. S6.** C-NMR spectrums of preparated benzamide.
